# Dysbarism: An Overview of an Unusual Medical Emergency

**DOI:** 10.3390/medicina58010104

**Published:** 2022-01-10

**Authors:** Gabriele Savioli, Claudia Alfano, Christian Zanza, Gaia Bavestrello Piccini, Angelica Varesi, Ciro Esposito, Giovanni Ricevuti, Iride Francesca Ceresa

**Affiliations:** 1 Emergency Medicine and Surgery, IRCCS Fondazione Policlinico San Matteo, 27100 Pavia, Italy; claudia.alfano86@gmail.com; 2 PhD School in Experimental Medicine, Department of Clinical-Surgical, Diagnostic and Pediatric Sciences, University of Pavia, 27100 Pavia, Italy; 3 “Ospedale Alba-Bra Onlus”—Department of Emergency Medicine, Anesthesia and Critical Care Medicine, Michele and Pietro Ferrero Hospital, 12060 Verduno, Italy; christian.zanza@live.it; 4 Research Training Innovation Infrastructure, Research and Innovation Department, Azienda Ospedaliera SS Antonio e Biagio e Cesare Arrigo, 15121 Alessandria, Italy; gaia.bavestrellopic01@universitadipavia.it; 5 Emergency Medicine, Université Libre de Bruxelles, 1000 Brussels, Belgium; 6 Department of Biology and Biotechnology, University of Pavia, 27100 Pavia, Italy; angelicavaresi@universitadipavia.it; 7 Unit of Nephrology and Dialysis, ICS Maugeri, University of Pavia, 27100 Pavia, Italy; ciro.esposito@unipv.it; 8 School of Pharmacy, Department of Drug Sciences, University of Pavia, 27100 Pavia, Italy; giovanni.ricevuti@unipv.it; 9 Emergency Department, Ospedale Civile Vigevano, 27029 Vigevano, Italy; irideceresa@gmail.com

**Keywords:** dysbarism, acclimatization, decompression illness, barotrauma, era barotrauma, sinus barotrauma, pulmonary compression barotrauma, decrease barotraumas, inert gas narcosis, gas embolism, oxygen toxicity, emergency medicine, hostile environmental medicine

## Abstract

Dysbarism is a general term which includes the signs and symptoms that can manifest when the body is subject to an increase or a decrease in the atmospheric pressure which occurs either at a rate or duration exceeding the capacity of the body to adapt safely. In the following review, we take dysbarisms into account for our analysis. Starting from the underlying physical laws, we will deal with the pathologies that can develop in the most frequently affected areas of the body, as the atmospheric pressure varies when acclimatization fails. Manifestations of dysbarism range from itching and minor pain to neurological symptoms, cardiac collapse, and death. Overall, four clinical pictures can occur: decompression illness, barotrauma, inert gas narcosis, and oxygen toxicity. We will then review the clinical manifestations and illustrate some hints of therapy. We will first introduce the two forms of decompression sickness. In the next part, we will review the barotrauma, compression, and decompression. The last three parts will be dedicated to gas embolism, inert gas narcosis, and oxygen toxicity. Such an approach is critical for the effective treatment of patients in a hostile environment, or treatment in the emergency room after exposure to extreme physical or environmental factors.

## 1. Introduction

Dysbarism is a general term which includes the signs and symptoms that can manifest when the body is subject to an increase or a decrease in the atmospheric pressure which occurs either at a rate or duration exceeding the capacity of the body to adapt safely [1,2].

The risk of developing dysbarism is higher than in the past decades, due to the advancement of technology and the spread of certain sports practices. Nevertheless, it still represents a medical emergency that is not widely researched.

Although the most common cause of dysbarism is underwater diving, it can also be associated with aviation and space exploration, as well as compressed air tunnel and caisson work. Furthermore, dysbarism can arise from iatrogenic causes unrelated to decompression, such as non-invasive ventilation and invasive mechanical ventilation.

Different pathologies can occur when the body is subjected to a pressure higher than the atmospheric one or when, after being subjected to it, it returns to normal pressure (decompression) too quickly. In the first case, they will develop when the pressure increases to the point of overcoming the adaptive mechanisms of the organism; in the second case, they will develop when the decompression time is too short for the adaptive mechanisms to be efficient.

Manifestations of dysbarism range from itching and minor pain to neurological symptoms, cardiac collapse, and death [3]. Overall, five clinical pictures can occur: decompression illness, barotrauma, gas embolism, inert gas narcosis, and oxygen toxicity.

In this review, we schematically introduce and define each one of them, and then briefly summarize their main characteristics, with a review of their pathophysiology, clinical manifestations, and therapy.

## 2. Classification of Dysbarisms

### 2.1. Decompression Illness

Decompression illness is a multisystem disease caused by the formation of gas bubbles in the blood and tissues during or after a decrease in environmental pressure (decompression). As the ambient pressure increases, the air pressure of the inert gas (generally N_2_) is greater than its arterial pressure, which causes the tissues to absorb this inert gas until the balance between the two pressures is reached. The absorption depends on the gradient tissue alveolus and on the blood–issue solubility ratio.

When a critical amount of nitrogen dissolves in the tissues, a rapid decrease in pressure causes the dissolved nitrogen to return to its gas form while still in the blood or tissues, causing bubbles to form [4]. These bubbles can have direct and indirect effects. The direct effects are represented by the mechanical obstruction of a vein or an artery. The indirect effects are mediated by an inflammatory response with activation of complement and coagulation factors and platelets. The result will be the formation of thrombi around the bubble, followed by an increase in vascular permeability, and consequent interstitial oedema with stasis of the microcirculation and tissue hypo perfusion up to ischemia.

The direct mechanism is also known as “arterial (or venous) gas embolism” (AGE), whereas the indirect mechanism is also known as “decompression sickness” (DCS).

Golding et al. proposed a classification of DCS into two forms: a minor form (Type I), in which the musculoskeletal system is affected, and a more severe form (Type II), which involves the neurological, pulmonary, and cardiac systems [5].

### 2.2. Barotrauma

Barotrauma is caused by a difference in pressure between a gas inside, in contact with, or outside the body, and the pressure of the surrounding gas or fluid [6]. It can occur either with an excessive increase (e.g., “downward” dive) or with a rapid decrease (e.g., “uphill” dive) of the external pressure. As external pressure increases, the gas volume in the body cavities containing air decreases (e.g., lungs, middle ear, sinuses, gastrointestinal tract, etc.). The opposite occurs when external pressure decreases. Tissue damage can result from pressure difference when the pressure in these organs does not equal the new environmental pressure.

Intubation and mechanical ventilation are common therapeutic maneuvers in anesthesia or in the ICU setting, and consists of applying positive pressures to the patient. This can be a cause of barotrauma.

Risk factors for the iatrogenic barotrauma include advanced age, malignancy of the upper or lower airway, and severe underlying lung disease affecting the alveoli (such as chronic obstructive pulmonary disease or COPD, acute respiratory distress syndrome or ARDS, acute lung injury or ALI, necrotizing infections, or COVID infection) [7,8,9,10].

Preexisting vascular pathologies (such as cerebral venous thrombosis; intracranial aneurysms; and diseases affecting the vertebral, carotid, or coronary arteries) may influence the patients’ onset [11,12,13,14,15,16,17].

Furthermore, all factors predisposing to hyperinflation, such as high transpulmonary pressure (airway pressure minus the pleural pressure), high tidal volumes, and high intrinsic PEEP, increase the risk of iatrogenic barotrauma.

The iatrogenic barotrauma, also known as “ventilator associated lung injury (VALI)”, or “ventilator-induced lung injury (VILI)”, can be subdivided into macrobarotrauma (the form of radiologically detected barotrauma) and microbarotrauma, with diffuse lung injury and possible injury of other organs due to release of inflammatory mediators’ biotrauma.

### 2.3. Gas Embolism

Gas embolism occurs when a pressure gradient allows air to enter the blood stream. This, in turn, causes a blockage in a blood vessel by one or more bubbles of air or other gas [18].

It constitutes a fearful clinical form, which can present itself as a complication of both barotrauma and decompression sickness or complicate the mixed forms.

### 2.4. Inert Gas Narcosis

Inert gas narcosis is the physical and mental disturbance, both subjective and objective, that occurs when breathing gas mixtures containing certain inert gases under pressure [19].

Breathing air at pressures higher than 4 ATA (100 fsw) leads to inert gas narcosis, due to the increased partial pressure in the central nervous system of inert gases (such as nitrogen).

### 2.5. Oxygen Toxicity

Breathing oxygen at higher-than-normal partial pressure can cause oxygen toxicity or oxygen poisoning [20]. Oxygen at partial pressures above 1.4 ATA can produce acute neurotoxicity.

The clinical settings in which oxygen toxicity occurs is predominantly divided into two groups: one where the patient is exposed to very high concentrations of oxygen for a short duration, and the second where the patient is exposed to lower concentrations of oxygen but for a longer duration. These two cases can result in acute and chronic oxygen toxicity, respectively [21].

Risk factors for developing oxygen toxicity include hyperbaric oxygen therapy, exposition to prolonged high levels of oxygen, prematurity, and diving.

## 3. Epidemiology of Barotrauma

Barotrauma of the ear, nose, and throat are nowadays the most frequent accidents during diving. ENT barotrauma affects 10% of dives with experienced divers and 30% with novice divers [22].

Arterial gas embolism, on the other hand, is usually precipitated by rapid ascent, breath holding, or the presence of lung disease. Thus, it is rare and has an apparently decreasing incidence.

The proportion of cases of decompression illness attributable to arterial gas embolism in recreational divers declined from 18% in 1987 to 8% in 1997 [23].

Of 441 confirmed or possible incidents of decompression illness in recreational divers reported to the Divers Alert Network, only 3.9% were classified as possible arterial gas embolism [24]. If appropriate decompression procedures are followed, decompression sickness is also uncommon. The rate of occurrence (per dive) in operational open water dives from minutes to several hours in duration varies according to the diving population: 0.015% for scientific divers, 0.01–0.019% for recreational divers, 0.030% for US Navy divers, and 0.095% for commercial divers [25,26].

The number of active worldwide recreational divers is not known but is likely to be in the millions. The Divers Alert Network took a sample of 135,000 dives by 9000 recreational divers in which the rate of occurrence of decompression sickness was 0.03%. This rate was much higher during dives in cold water than during dives in warm water [24]. These numbers are all based on many dives made well within the maximum exposure limits of accepted procedures (decompression tables or computers) and, therefore, are underestimates of the true rates at the maximum limits. For example, the rate of occurrence of decompression sickness for US Navy dives from 1971 to 1978 at the maximum limits was 1.3% [27].

Moreover, for long exposures under stressful thermal and exercise conditions, the US Navy dive trials aimed to develop new decompression procedures with an occurrence rate of 4.4 cases of decompression sickness per 100 dives [28].

## 4. Decompression Sickness: From Physiopathology to the Clinic, from the Clinic to Therapy

### 4.1. Physiopathology

**Figure 1:**
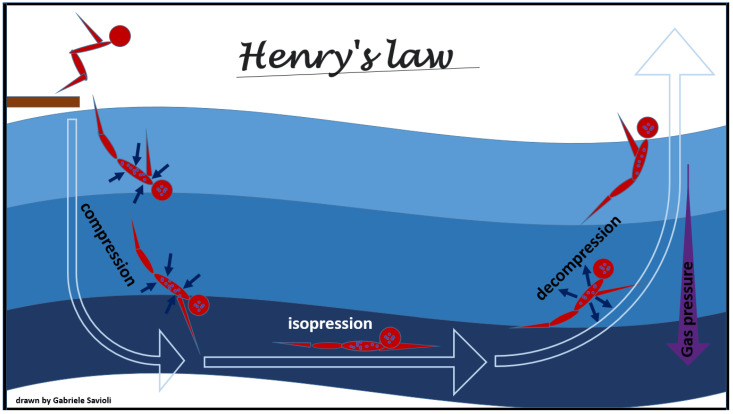
Henry’s law.

The physical law that mostly underlies adaptive changes and decompression pathology development and on their overcoming is Henry’s law. This law, formulated by William Henry in 1803, states that “a gas which exerts a pressure on the surface of a liquid enters into solution until it reaches the same pressure in that liquid as it exerts on it”. According to this law, at constant temperature, the solubility of a gas is directly proportional to the pressure that the gas exerts on the solution (Figure 1).

Once the equilibrium is reached, the liquid is defined as saturated with that gas. As the pressure increases, another gas will enter the solution, while as it decreases, the liquid will find itself in a situation of supersaturation and the gas will be released returning to the outside until the pressures are again balanced.

For this principle, when the environmental pressure is reduced during decompression, the tissues become supersaturated with inert gases, and, therefore, the gases tend to leave the solution and form free gas. Due to the metabolic activity of oxygen and carbon dioxide, oxygen and carbon dioxide saturation rarely contributes to phase formation [29,30].

Since the gas transfer into tissues is a dynamic process, it takes time to reach the equilibrium between the tissues and the environmental partial pressure of the inert gas. Some time is needed in order to reach an equilibrium of the tissue gas concentration at a given pressure. The inert gas which escapes from a tissue follows a similar kinetic pattern when the ambient pressure is reduced. Boycott has shown that staged decompression with set breaks could minimize the degree of supersaturation [31,32]. Mathematical models on the different types of tissues and gases have been developed to describe these steps, and useful tables have been created to determine decompression times.

It should be noted that, because of this principle, reaching certain altitude levels (e.g., flying in a commercial aircraft) within 12–18 h after diving can result in the formation of free gas in the tissues, even following the protocols established for safe decompression.

When returning to atmospheric pressure from a situation of increased surrounding pressure, the organism which now finds itself in a condition of nitrogen supersaturation requires adequate time to dispose of this inert gas. The inert gas which was taken with the breathed air has, indeed, solubilized in the tissues, especially in those with lipid content (adipose tissue and myelin sheaths), in quantities directly proportional to environmental pressure and time of exposure.

For the calculation of the time necessary for the clearance of the nitrogen supersaturated quota without biological damage, decompression tables are elaborated on biological mathematical models. Failure to respect these times determines the pathogenetic moment of this syndrome. Nitrogen is released in the gaseous phase, forming bubbles in the cellular environment, in interstitial liquids, and in the circulation, where it can cause embolism. At the interface with interstitial liquids and plasma, the bubbles can also indirectly activate the intrinsic pathway of coagulation, platelet aggregation, and factors responsible for the cascade of inflammation.

It is important to highlight that DCS also occurs when the decompression profile is executed, suggesting individual vulnerabilities that go beyond the mathematical aspects of the calculation. An interesting European study found that most of the dives took place in the “safe zone”, even if the data show a clear “gray area” to the “mathematical” ability to predict DCS via current algorithms. Hence, there is a need to find new algorithms. The study shows that several other risk factors appear to affect the likelihood of developing DCS, regardless of their effects on the bubble formation and some factors that affect or increase the effects of the bubble formation. The authors also suggest considering risk factors such as BMI, fat mass, stress response, and hormonal cycles, thus opening up new avenues for research [33].

### 4.2. Clinical Forms

Decompression sickness (DCS) has an acute onset; however, the time of onset is variable from individual to individual and, although generally the signs and symptoms arise within two hours after an activity conducted in an environment with higher pressure than the atmospheric one (hyperbaric), it can be of a few minutes up to 24–48 h.

Most cases of decompression sickness occur soon after surfacing, with 98% of cases occurring within 24 h; however, in some rare cases, the clinical picture may appear after 48 h [32]. This situation is typical of scuba diving carried out with self-contained breathing apparatus (ARA or SCUBA-AIR). Decompression sickness may also occur following exposure to high pressures or even following the rapid loss of pressure in the cabin of an aircraft.

**Figure 2:**
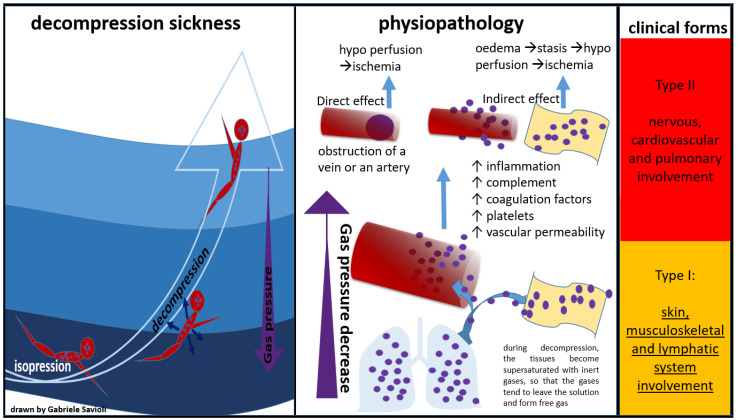
Physiopathology of decompression sickness.

Bert was the first to describe DCS pathophysiology in his milestone “La Pression Barometrique”, published in 1878 [34]. Subsequently, in the early 20th century, autopsy studies on divers and caisson patients suggested that DCS was due to free gas in the blood and tissues. Hallenbeck was then the first to demonstrate that platelet activation, intravascular coagulation, and impaired capillary permeability (with plasma leakage into the extravascular space) were all correlated with bubble surface activity (Figure 2) [35].

DCS occurs in about 1 in 5000 dives for the professional diver (a risk comparable to that of military divers) while amateurs are the category at highest risk [36,37].

According to the Golding classification, we can divide DCS into two categories.

#### 4.2.1. Type I DCS: Skin, Musculoskeletal, and Lymphatic System Involvement

It is the most common type of DCS and causes joint pain that is often confused with pain from injuries [38,39]. It is the least severe form of DCS, in which no neurological, cardiovascular, or respiratory symptoms appear. The patient may complain of general malaise, asthenia, and fatigue. The most common manifestation remains; however, the joint pain, which derives from the fact that the movements of the bone heads can cause negative pressure, therefore attracts gas bubbles. Shoulders and elbows are generally the most frequently involved joints. Myalgia with variable localization is also frequent, and it is the expression of the activation of the cascade of inflammation, mediated by the nitrogen bubbles. The symptoms of musculoskeletal involvement may disappear within a few hours or persist for 4–5 days. A history of prior musculoskeletal DCS increases the risk of osteonecrosis. Osteonecrosis can occur in divers who experienced deep and prolonged exposure in caisson [39], diving instructors, and commercial divers [40]. Additional and more rare presentations involve the cutaneous and lymphatic system, which led to itching, marbling, and skin edema with an orange peel appearance. A particular skin involvement is cutis marmorata, which usually manifests itself with the form of itchy or painful reddish bluish discoloration, generally considered mild decompression sickness (DCS), and relies on high-pressure recompression therapy. It is treated with oxygen inhalation. The appearance of patent foramen ovale has been observed to be associated with the presence of patent foramen ovale (PFO) in the heart, with a prevalence almost 100%. Cutis marmorata rarely has other DCS symptoms. These symptoms usually take the form of blurred vision and dizziness, as well as mild vague or systemic brain dysfunction (abnormal malaise, clumsiness, poor concentration, etc.). The etiology of these other symptoms is clearly embolic, and skin marmorata may also be a symptom of gas vesicles that embolize the brainstem. The regulatory sites of skin blood vessels for dilation and contraction of the skin by the autonomic nervous system [41,42,43,44,45].

#### 4.2.2. Type 2 DCS: Nervous, Cardiovascular and Pulmonary Systems Involvement

It is the most severe form, albeit less frequent, and can cause permanent damage and, in rare cases, death. The spinal cord is the most common site affected by type II DCS. Symptoms mimic spinal cord trauma and usually involve the lumbar or lower dorsal level of the spine [46]. The onset is often characterized by paresthesia and strength deficits up to paraplegia, neurological bladder, bowel or bladder incontinence, and sexual impotence [47]. When DCS affects the brain, many symptoms can follow, and the clinical picture may be dominated by ataxia, nystagmus, visual disturbances, language disorders, behavioral changes, seizures, and even a coma. These manifestations are usually secondary to deep and prolonged diving and often result in permanent deafness [48,49].

Cardiac or respiratory symptoms begin with retrosternal oppression or pain associated with cough or dyspnea. In some cases, bronchospasm may develop. The alterations that occur instead, in the case of embolization of the pulmonary circulation, are typical of acute pulmonary heart and right heart failure. In some cases, right heart failure can lead to coma or death. The embolization of coronary arteries or large cardiac cavities can also lead to cardiac arrest.

Pulmonary vascular obstruction usually occurs when a massive quantity of gas transits in the venous system. Clinically speaking, this results in chest pain, dyspnea, and cough [50]. This presentation occurs in about 2% of all DCS cases and can eventually cause death. Symptoms can start up to 12 h after a dive and can persist for 12–48 h. Pulmonary barotrauma may be associated to type 2 DCS with pulmonary involvement in the case of rapid ascent (see below).

### 4.3. Therapy

Since the presence and detection of gas emboli is usually the first evidence of free gas, the study of the pulmonary artery with thoracic CT scan can be used as detection tool; furthermore, ultrasound can also be useful [51].

For forms with immediate onset, the first intervention may take place at the scene of the decompression accident and primarily includes the support of vital functions, in accordance with the usual guidelines for cardiopulmonary resuscitation. Oxygen needs to be administered as soon as possible with an oronasal mask at FiO_2_ = 1, and the absence of nitrogen in the mixture favors its elimination by the body [52,53].

The administration of intravenous or oral fluids for conscious patients to expand the volume and improve blood rheology should be considered from the very beginning.

During hospitalization, the measures already described may be integrated with the administration of non-steroidal anti-inflammatory drugs, useful in the arthromyalgic manifestations [54]. In fact, some studies suggested that additional interventions, such as non-steroidal anti-inflammatory drugs (NSAIDs) or recompression with helium in addition to oxygen, could reduce the recompression time required. For example, using NSAIDs reduced the average number of recompressed sessions required from three to two. Using any of these strategies may be justified. A modest number of patients studied require careful interpretation. Further research is needed [54,55,56]. Antiplatelet agents, such as aspirin, can be given both to counteract platelet activation caused by free gas in the blood, and for their ability to antagonize the increase in platelet and erythrocyte aggregation [53,57].

In the case of neurological bladder development, a bladder catheter needs to be put in place.

The transport of patients to centers equipped with hyperbaric implants must be prepared quickly. The use of air vehicles requires the use of pressurized cabins or low-altitude flights in order to avoid further decompression of the patient.

**Figure 3:**
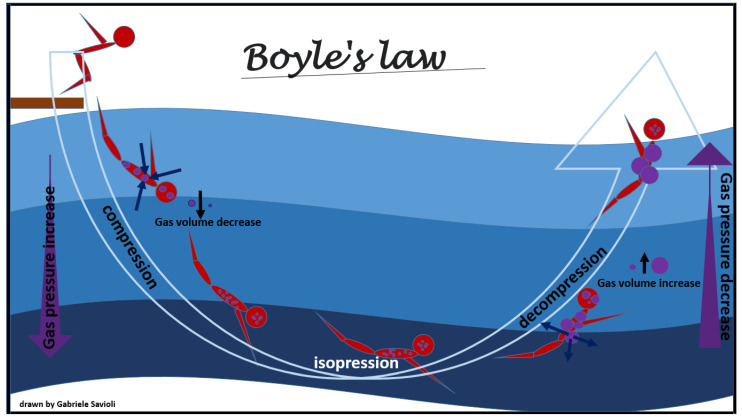
Boyle’s law.

Therapeutic recompression, meaning administration of 100% oxygen for several hours in a sealed chamber pressurized to >1 atmosphere, gradually lowered to atmospheric pressure, may be needed, and has three main purposes [58]:

- To reduce the volume of bubbles present in the body, according to Boyle’s law (Figure 3), and, therefore, to reduce the embolic obstacle to the bloodstream. This will reduce the total surface of the bubbles with consequent reduction in the interface with interstitial liquids and plasma and, in turn, reducing the activation of coagulation, platelet aggregation, and inflammation processes;
- To increase absorption of the bubbles in the body fluid and exhalation of inert gases from the lung, through solubilization of the nitrogen present in the bubbles (Henry’s Law, Figure 1);
- To increase oxygen supply to the cells in peripheral tissues.

## 5. Barotrauma

### 5.1. Physiopathology

Barotrauma is physical tissue damage caused by a pressure difference between an unvented space inside the body and surrounding gas or fluid. This, in turn, causes an expansion or compression of gases in gas-filled organs. The damage in barotrauma is, therefore, due to compressive/expansive forces and shear, leading to overstretching of tissues [59].

As reported in the pathophysiology of trauma, the barotrauma can affect several organs [60,61,62,63,64,65,66,67,68,69]. The most frequent injuries involve the sinuses and/or middle ear, but barotrauma may also cause facial, tooth, gastrointestinal (GI), pulmonary (pneumothorax, pulmonary hemorrhage), and mediastinal injuries. In addition, facial nerve injuries have also been reported [60,61].

The site of injury varies according to whether the barotrauma is caused by an increase or a decrease in pressure. The organs most frequently affected by an increase in pressure are the ear, the paranasal sinuses, and the lungs. The organs most frequently affected by a decrease in pressure are the lung, the ear, the mediastinum, and the gastrointestinal tract.

The physical law most involved in adaptive changes and in the development of this pathology is Boyle’s law (Figure 3). Boyle’s law states that, at a constant temperature, the pressure of an ideal gas is inversely proportional to its volume, i.e., when the product of the gas pressure by the volume it occupies is constant [70]. Therefore, when the absolute pressure of the gas is doubled, its volume is reduced to half of the original volume.

According to this physical law, the volumetric change due to the passage from 2 to 3 absolute atmospheres (ATA) is less than the volumetric change from 1 to 2 ATA; in other words, for a given variation in depth, the change in the volume of the gas is greater the closer a person is to the surface.

### 5.2. Clinical Forms and Respective Therapies of Pressure Increase Barotraumas

#### 5.2.1. Pulmonary Compression Barotrauma

**Figure 4:**
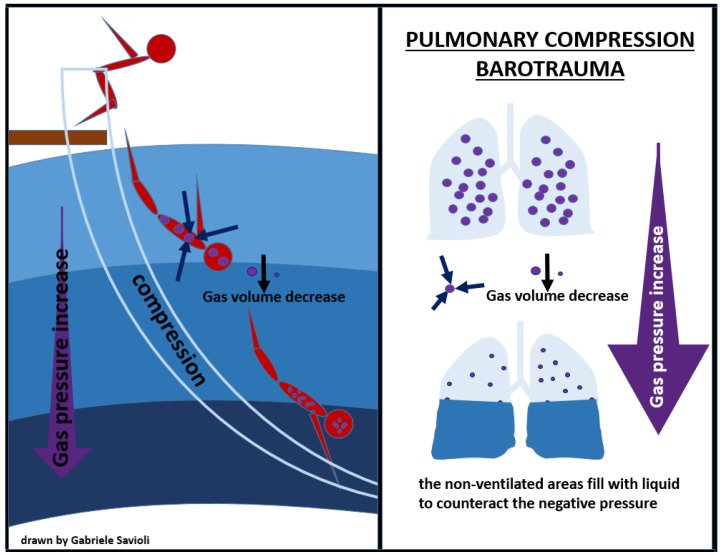
pulmonary compression barotrauma.

Compression of the lungs may occur during very deep descent in breath-hold diving and may cause the lung volume to decrease at a level equal or below the residual volume, causing mucosal edema, vascular engorgement, pulmonary edema, and hemorrhage, which can clinically manifest as chest pain, dyspnea, and hemoptysis on ascent (Figure 4) [71].

The treatment of pulmonary compression barotrauma involves administration of 100% O_2_, fluid infusion, and supportive therapy, according to clinical conditions.

Inherent lung damage with alveolar rupture can allow air into the pulmonary venous circulation and can cause air embolism; therefore, positive pressure ventilation, in both Bi-PAP and C-PAP modes, is contraindicated [71,72].

#### 5.2.2. Ear Barotrauma

**Figure 5:**
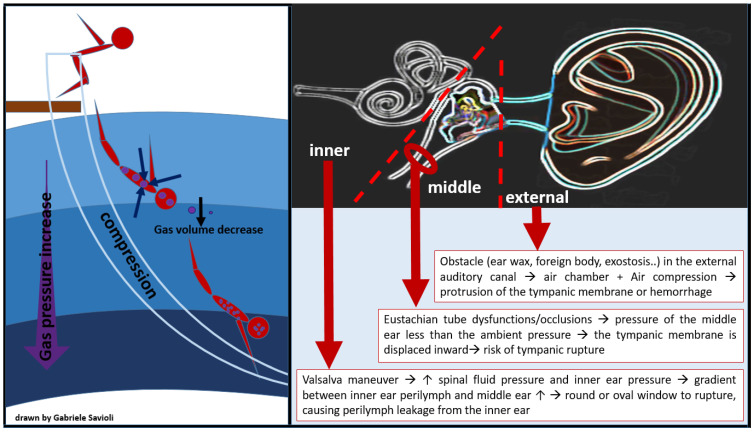
Barotitis: external, middle, and inner.

- External ear barotrauma occurs when the presence of an obstacle in the external auditory canal (ear wax, foreign body, exostosis), leading to an air chamber between the eardrum and the water. The air compression which derives from the increase in pressure causes external protrusion of the tympanic membrane and eventually hemorrhage (Figure 5). External ear barotrauma manifests itself with ear pain or bloody otorrhea.
- Middle ear barotrauma occurs when the middle ear fails to compensate for the external environmental pressure. The eustachian tube (ET), which connects the middle ear space to the throat is, in normal conditions, responsible for the exchange of air between the nasopharynx and the middle ear space, maintaining equal pressure between the middle ear and the external auditory canal [73]. Risk factors for developing middle ear barotraumas are, therefore, the presence of anatomical variant-dependent Eustachian tube dysfunctions/occlusions, such as mucosal congestion (due to upper respiratory tract infections, allergy, smoke), presence of polyps, too-deep self-inflation maneuvers, and maxillary facial trauma. As the ambient pressure (atmospheric or hydrostatic) increases externally to the body, it causes pressure to rise in the external auditory canal. While the gas volume in the middle ear space decreases, the tympanic membrane is, therefore, displaced inward, causing ear pain and creating a vacuum. In order to compensate for this decrease in gas volume, equalization of the middle ear space pressure is required, for example through a Valsalva maneuver during descent in divers. When the middle ear space is not filled sufficiently with air from the nasopharynx, middle ear barotrauma occurs. Importantly, as the pressure gradient rises with increasing depth, the risk of tympanic rupture also increases (Figure 5) [70].
- Inner ear barotrauma represents the most severe form of ear injury from barotrauma and can have permanent consequences. Compression barotrauma of the inner ear occurs when, as a diver descends and the surrounding pressure increases, the tympanic membrane is pressed medially. This will, in turn, press the stapes into the oval window, causing increased pressure in the cochlea and bulging of the round window. At a pressure differential of >90 mmHg (12 kPa), however, the Eustachian tube will ‘lock’ closed, preventing a successful Valsalva [74,75]. Repeated increasingly forceful Valsalva maneuvers will, in consequence, increase spinal fluid pressure and inner ear pressure. The gradient between the inner ear perilymph and the middle ear can eventually become large enough to cause the round or oval window to rupture, causing perilymph leakage from the inner ear (Figure 5) [70]. When inner ear barotrauma occurs, the diver experiences q sudden onset of dizziness, tinnitus, and hearing loss. Nausea and vomiting can result from vestibular involvement. Treatment may require surgical repair and, if left untreated, hearing loss or tinnitus can become chronic.

#### 5.2.3. Sinus Barotrauma

The paranasal sinuses communicate with the nasal cavity via small orifices (ostia), which can, in some circumstances, be blocked by inflammatory processes, such as upper respiratory tract infections or allergies. Ostia blockage can impair sinus drainage and the paranasal sinuses can, therefore, fail to equalize to barometric changes during vertical travel [76,77]. Failure to compensate for air pressure in the sinuses during descent causes the pressure inside the sinuses to become lower than the ambient pressure. When ambient pressure exceeds the pressure in the sinuses, engorgement and vascular rupture occur, causing pain at the level of the sinuses on descent and epistaxis on ascent.

In addition, persistence of blood in the sinuses can subsequently cause bacterial sinusitis.

During ascent, compression of the maxillary branch of the trigeminal nerve flowing into the maxillary sinuses can also occur, causing an infraorbital paresthesia that, without treatment, resolves within several hours [78].

#### 5.2.4. Additional Minor Forms of Barotrauma

Failure to compensate for the air in the diving mask during descent can cause facial barotrauma. It is possible to find edema and bruising where the diving mask rested and conjunctival hemorrhages appear. Retroorbital hematoma and diplopia have also been described. No therapy is needed for these minor forms of barotrauma.

### 5.3. Clinical Forms and Respective Therapies of Pressure Decrease Barotraumas

**Figure 6:**
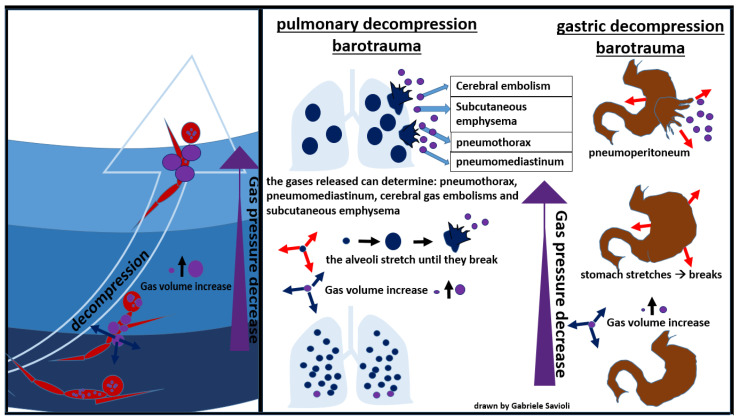
Decompression barotraumas.

As the pressure decreases, the volume occupied by the gases increases (Boyle’s law, Figure 3), and the excess of gas is exhaled. If the surplus is retained, the tissues that contain gas are stretched and damaged (Figure 6).

#### 5.3.1. Pulmonary Decompression Barotrauma

Pulmonary decompression barotrauma, first described in 1932 by Behnke and Polak and Adams, represents the most serious form of dysbarism [79,80].

**Figure 7:**
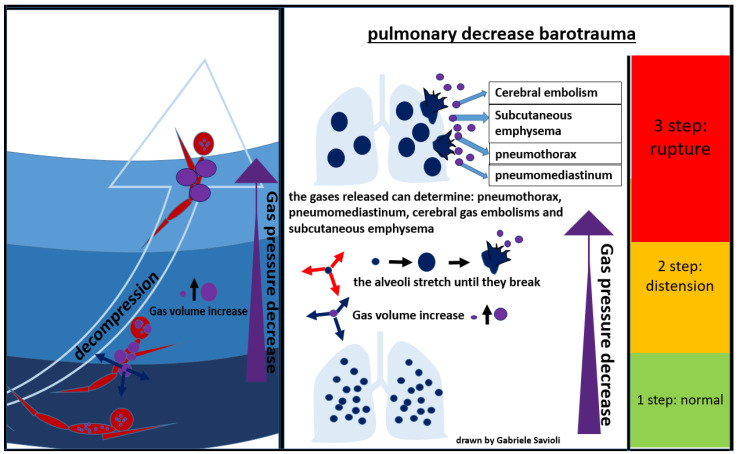
Pulmonary decompression barotrauma.

During an activity conducted in an environment with a pressure higher than the atmospheric one, the inhaled air is constituted by a mixture of gases at the same pressure as the surrounding environment. As the pressure decreases, as it occurs, for example, during the ascent to the surface in the case of divers, the gases expand according to Boyle’s law. If the surplus of air is not exhaled, alveolar expansion occurs, which may lead to alveolar rupture, with gas spreading in the surrounding tissues (Figure 7) [81].

In the presence of high interalveolar pressure, the gas can be forced into the pulmonary capillaries and arterial circulation, provoking gas embolism (Figure 7).

Gases can then enter the mediastinum and pleural space, producing pneumomediastinum and pneumothorax respectively [81]. Pneumomediastinum can complicate pulmonary barotrauma in about 25% of cases [82]. Pneumothorax is less frequent (3%) but often severe, as the evolution towards hypertensive pneumothorax is frequent.

The most serious complication of pulmonary barotrauma derives from cerebral embolization, resulting in memory loss, hemiplegia, confusion, visual disturbances, seizures, dizziness, or headaches.

Other frequent complications are subcutaneous emphysema (10%), pneumopericardium (6%), (3%), pneumoperitoneum (3%), and pulmonary aspiration (Figure 7) [82]. Cardiac arrest can occur in about 5% of cases.

The clinical picture in pulmonary decompression barotrauma is dominated by a sensation of chest weight, chest pain, cough, and dyspnea.

The diagnosis of pulmonary decompression barotrauma and its complications is based on imaging with a CT scan if available, or a chest ultrasound or chest X-ray.

The treatment of pulmonary compression barotrauma involves administration of 100% oxygen therapy and liquid infusions.

Decompression maneuvers, such as chest drain placement or decompression mediastinotomy, should be considered. Recompression in a hyperbaric chamber may be necessary.

#### 5.3.2. Gastrointestinal Decompression Barotrauma

Gastrointestinal decompression barotrauma is caused by the expansion of intestinal gas after an activity in an environment with a pressure higher than the atmospheric one, when the subject returns to lower pressure.

Factors that seem to increase the risk of developing this form of barotrauma include previous gastric surgeries, aerophagia, and intake of carbonated beverages [83].

The clinical picture, in this case, is dominated by symptoms which occur due to gastric distension, including abdominal colic, belching, flatulence, and syncope (due to a vagal reaction).

**Figure 8:**
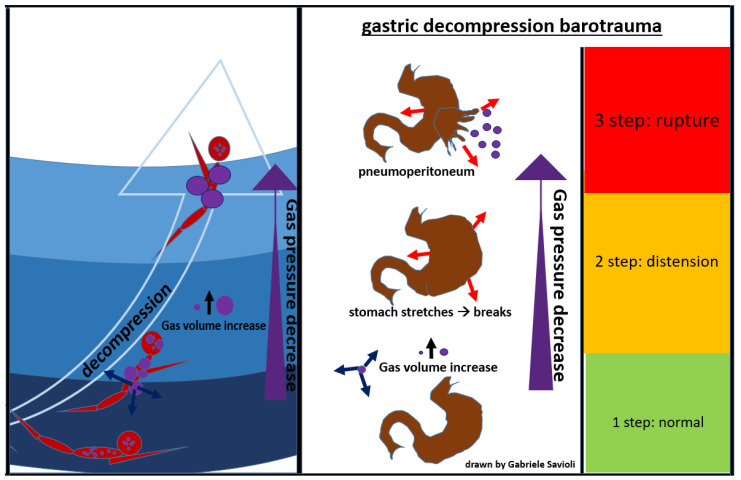
gastric decompression barotrauma.

A rare but fearful complication is gastric rupture, resulting in pneumoperitoneum (Figure 8) [84].

Gases from the esophagus, when expanding due to a decrease in surrounding pressure, can also cause pneumoperitoneum. Gastrointestinal decompression barotrauma requires a surgical treatment.

## 6. Gas Embolism

### 6.1. Pathophysiology

Arterial gas embolism (AGE) results from one of three mechanisms:

- direct bubble embolization after pulmonary barotrauma causes alveolar rupture, mainly as a complication of pneumothorax. In this case, when lung tissue tears, gas bubbles enter the pulmonary veins, and, from there, they can migrate to the systemic circulation;
- right to left shunt through a patent foramen ovale. The predisposing factors to developing gas embolism through this mechanism are chronic obstructive pulmonary pathologies and presence of right left shunt at a cardiac or vascular level;
- passage of bubbles to the systemic arterial circulation when the venous bubble burden is too important for the pulmonary capillary beds to clear the gas into the alveoli fast enough. Such bubbles may form either as a result of barotrauma or as a result of decompression sickness.

### 6.2. Clinical Forms

The symptoms of gas embolism are sudden and striking, and arise within the first ten minutes, and more frequently within the first two, from the end of the activity carried out at a pressure higher than the atmospheric one.

At the systemic level, the gas bubbles occlude arterioles smaller than their own. The clinical manifestations, therefore, depend on the size of the bubbles and the site of the occlusion.

Frequently, the pathology involves the cutaneous district with cyanosis or pallor of the ischemic region. The tongue is frequently affected.

The organs most sensitive to bubbles remain, however, those with the most obvious resulting signs and symptoms, that is, the central nervous system and coronary arteries. As a result, AGE often causes symptoms similar to acute coronary syndromes and stroke syndromes [85,86].

At the level of the cerebral circulation, gas emboli can cause motor or sensory deficits, convulsions, loss of consciousness, severe alterations in breathing, and eventually death.

At the level of the coronary circulation, gas emboli can cause acute coronary syndrome, myocardial infarction, arrhythmias, and cardiac arrest [87].

Furthermore, gas embolism of the small circulation associated with the presence of pneumothorax with displacement of the mediastinal structures can lead to a reduction in cardiac output and clinical pictures of shock. Even less frequently, the involvement of the digestive tract has been described.

### 6.3. Therapy

The first therapeutic measure for the treatment of gas embolism is the administration of 100% oxygen to facilitate the reabsorption of the gas bubbles in the blood. Hydration can help improve perfusion at the level of the ischemic districts. The patient should be transported to the hospital as quickly as possible, in a supine position, in order to reduce the risk of cerebral embolization. In the case of persistent ischemia, intermittent and repeated sessions of the hyperbaric chamber are indicated during hospitalization.

## 7. Inert Gas Narcosis

### 7.1. Pathophysiology

Multiple theories have been proposed to explain the pathophysiologic mechanism of inert gas narcosis [19].

Breathing compressed air while at atmospheric pressures greater than 1 ATM increases the partial pressures of nitrogen and oxygen, in the blood. The effect of nitrogen on the body takes place in the central nervous system (CNS), but the exact site and mechanism are still debated [88].

Inert gas narcosis is thought to be caused by a rise in partial pressure levels of nitrogen at the level of the central nervous system. As the external pressure increases, the partial pressure of nitrogen dissolved in the blood rises, significantly increasing the possibility of binding to oxygen, and forming dinitrogen oxide (N_2_O), an analgesic and anesthetic also known as laughing gas, thus causing a toxic effect on the organism, known as nitrogen narcosis.

Inert gas narcosis, however, is not only linked to nitrogen and the formation of dinitrogen oxide, but also to other gases present in the mixture breathed underwater.

### 7.2. Clinical Forms

Inert gas narcosis usually occurs after diving at depths greater than 30 m [82]. The clinical presentation includes symptoms similar to alcoholic or benzodiazepine poisoning.

Nitrogen narcosis initially manifests through impairment of judgment, reasoning, short-term memory, and concentration. Further increases in the partial pressure of nitrogen in the blood from descending deeper lend to impairments in manual dexterity and further mental decline, including idea fixation, hallucinations, and, finally, stupor and coma [88].

Partial nitrogen pressure above 10–12 ATA (300–400 fsw) eventually produces an anesthetic effect, leading to unconsciousness. Death can, therefore, result from unconsciousness associated with severe narcosis or from severely impaired judgment, leading to an accident of some form during the dive.

Risk factors for the development of inert gas narcosis include alcohol consumption, fatigue, anxiety, and hypothermia.

### 7.3. Therapy

When inert gas narcosis is present in less severe forms, the decrease in altitude is sufficient for the complete remission of symptoms.

In the most severe forms, in which acute cardiorespiratory insufficiency has been established, it may be necessary to carry out cardiopulmonary resuscitation maneuvers to maintain vital functions.

## 8. Oxygen Toxicity

Oxygen neurotoxicity can occur following hyperbaric oxygen treatment or during scuba diving, especially when divers use breathing mixes with oxygen partial pressures higher than that of air.

In 1878, the French physiologist, Paul Bert, published “La Pression barométrique”, making him the first to demonstrate how high oxygen concentrations are toxic to many forms of life [34]. Oxygen at partial pressures higher than 1.4 ATA can indeed produce acute neurotoxicity [89], and this condition is directly dependent on the exposure time.

Central nervous system oxygen toxicity usually presents with prodromal symptoms, such as sweating, twitching, and visual changes (especially tunnel vision), which can be followed eventually by tonic–clonic seizures.

In some rare occasions, lung injuries may occur, resulting from an inflammation of the upper airways which then spreads to the lungs [41].

Oxygen toxicity is most commonly brief and resolves spontaneously once the partial pressure of oxygen is reduced.

## 9. Conclusions

Dysbarism occurs in a small percentage of the population, but it constitutes a widely diffused problem that few physicians are trained to recognize and manage.

Although its manifestations are often mild, permanent injury can occur in the most severe cases, especially when it goes unrecognized or when it is inadequately treated.

The emergency physician must be trained to suspect dysbarism on the basis of the clinical and medical history and to treat these conditions.
